# ‘I Thought I Could Cope Alone’: Understanding Barriers to Support‐Seeking Among Thyroid Cancer Survivors Through an Integrated Theoretical Lens

**DOI:** 10.1002/pon.70627

**Published:** 2026-10-24

**Authors:** Alicja Rogusz, Jamie Harding

**Affiliations:** ^1^ Faculty of Health Sciences and Wellbeing, University of Sunderland Sunderland UK; ^2^ Faculty of Society and Culture Northumbria University Newcastle upon Tyne UK

**Keywords:** cancer survivors, emotional well‐being, health services accessibility, help‐seeking behaviour, illness perception, patient acceptance of health care, psycho‐oncology, psychosocial support, survivorship care, thyroid cancer

## Abstract

**Background:**

Thyroid cancer has a favourable prognosis, yet many survivors experience ongoing psychological and social challenges. Although support is available, it is often underused. This study explored barriers to support‐seeking in the UK.

**Methods:**

An inductive, qualitative approach was taken. Twenty‐five adults with papillary or follicular thyroid cancer were recruited through an NHS Trust and a national charity. Semi‐structured interviews were conducted and thematically analysed, with the findings compared to existing bodies of theory.

**Results:**

Barriers clustered into two domains. Domain A focused on personal illness representations: (1) self‐sufficiency and low perceived need (‘I thought I could cope alone’), (2) altruistic reluctance (‘others need it more than me’), and (3) limited sense of belonging due to age or cancer type (‘not for people like me’). Domain B captured structural influences: (4) absence of proactive follow‐up (‘care ends at discharge’) and (5) support options that did not meet individual preferences (‘one‐size doesn't fit all’). An approach that combined the Common‐Sense Model of Illness Representations and Andersen's Behavioural Model of Health Services Use was most helpful in contextualising these findings. This approach showed how beliefs about coping, emotional regulation, and moral comparisons intersect with service design, information quality, and enabling resources to limit engagement with psychosocial support.

**Conclusion:**

Barriers arise through the interaction of belief‐based and system‐level factors. Clinically, routine assessment of illness perceptions, normalisation of help‐seeking, proactive survivorship follow‐up, and flexible, tailored support options may improve engagement. Embedding psychosocial care within standard follow‐up could help position emotional well‐being at the centre of thyroid cancer recovery.

## 1 Background

The thyroid, a small endocrine gland, regulates metabolism. While thyroid nodules are common, approximately 10% are malignant [1, 2]. The highest number of new thyroid cancer cases occurs in people aged 55–59 years [3]. Differentiated thyroid carcinomas (DTC), including papillary and follicular types, generally have favourable prognoses, though aggressive variants can occur unpredictably [4]. Recurrence is reported in 5%–20% of patients while distant metastases develop in 5%–23%, mainly in the lungs and bones [1]. Survivors, particularly those with micropapillary cancer, may face an increased risk of secondary malignancies, defined as new primary cancers that occur after the original thyroid cancer diagnosis [5].

Despite high survival rates, thyroid cancer (TC) significantly impacts long‐term quality of life [6, 7, 8]. Comparisons using the Quality of Life Cancer Survivor Instrument—which measures, physical, psychological, social and spiritual wellbeing—have suggested that survivors of TC have a quality of life comparable to or worse than that of patients with more aggressive cancers [9]. Research highlights significant unmet support and information needs among cancer patients, including those with thyroid cancer [10, 11, 12]. Healthcare providers may not routinely assess emotional well‐being and patients often hesitate to disclose distress [11]. Even when support is available, some patients opt out due to perceived self‐sufficiency or feelings of unworthiness [13].

This study offers a qualitative examination of the barriers TC survivors face when accessing psychosocial support in the UK. It helps to fill three gaps in the existing literature. Firstly, despite growing research on unmet needs in cancer survivorship, few studies have focused on TC, a condition often perceived as ‘less serious’, yet associated with persistent psychological and social challenges. Secondly, existing studies typically focus on quality of life or informational needs, with far less attention paid to the psychological and contextual factors that shape whether people engage with available support. Finally, few studies of patients of any cancer examine these influences using a coherent theoretical framework, and no UK‐based qualitative work has explored how personal illness beliefs interact with system‐level factors to affect help‐seeking.

## 2 Methods

### 2.1 Study Design and Methodological Orientation

The data discussed in this article was part of a wider, previously published project that used semi‐structured interviews to examine TC survivors' lived experiences of diagnosis, treatment, and follow‐up care [14]. Data was collected between 2019 and 2020. The present study is a secondary qualitative analysis of the same dataset, focusing specifically on barriers to support‐seeking. An inductive approach was taken to examining the data on seeking and accepting support, with themes being identified from interview transcripts and then a search being made of the existing literature for theories that best contextualised these themes [15].

### 2.2 Participant Recruitment and Sampling

Participants were recruited purposively through collaboration with an NHS Trust TC clinic and the Butterfly Thyroid Cancer Trust (BTCT) via their social media. Eligibility criteria required participants to be aged 18 years or older, to have a confirmed diagnosis of *papillary* or *follicular* thyroid cancer, and to be able to provide informed consent. A total of 25 patients took part in the study. Data was collected from eight participants prospectively: they were first interviewed within 6 weeks of diagnosis and then followed across their treatment trajectory. The remaining 17 participants had been diagnosed within the previous 5 years and contributed retrospective reflections on their adjustment and coping processes. Informed consent was obtained prior to participation, and all participants were informed of their right to withdraw at any stage. No remuneration was offered for participation.

### 2.3 Data Collection Procedures

Data were collected through in‐depth semi‐structured interviews designed to elicit participants' lived experiences of TC and its psychosocial impact. A semi‐structured interview guide was developed based on the study aims. It was reviewed after the first interview with a thyroid cancer survivor and refined iteratively throughout data collection to improve question clarity and explore emerging issues. These interviews lasted approximately 60–90 minutes, were conducted either face‐to‐face or remotely (via telephone or Microsoft Teams) in accordance with COVID‐19 safety guidance and relevant ethical approvals. The interviewer, an experienced clinician, created a supportive, non‐judgemental environment that facilitated open discussion of sensitive topics. Although no substantive differences emerged between prospective and retrospective accounts, the potential for recall bias in retrospective narratives was acknowledged and considered during analysis.

### 2.4 Data Analysis

The interview data was analysed using a reflective thematic approach, which is particularly well suited to inductive methodology [16]. Codes and themes arose from the transcripts, without any prior framework being imposed. Such an approach helps to identify how participants make sense of their own experience [17].

### 2.5 Ensuring Trustworthiness and Rigour

Several strategies were employed to enhance the trustworthiness of the findings. Credibility and analytical rigour were supported through verification of the data analysis by a second qualitative researcher, who independently reviewed the coding and emerging themes from five interview transcripts. The coding and thematic interpretations were then compared and discussed until agreement was reached, confirming consistency in the interpretation of the data. Reflexivity was maintained through the use of a reflective journal, allowing the researcher to acknowledge their clinical background and potential preconceptions, thereby enhancing transparency and credibility. Confirmability was strengthened by maintaining an audit trail documenting key analytical decisions throughout the study. Transferability was facilitated by providing rich, contextualised descriptions of participants' experiences and demographic characteristics, enabling readers to assess the applicability of the findings to other settings. Authenticity was enhanced through the inclusion of direct verbatim quotations that captured the emotional tone and nuance of participants' experiences.

### 2.6 Patient and Public Involvement

Patients, clinicians, and representatives from BTCT contributed to the study's design and implementation. Their insights shaped the recruitment strategy, interview schedule, and participant materials, ensuring that the research remained relevant, ethically sensitive, and accessible to the TC community.

### 2.7 Ethical Considerations

Comprehensive ethical safeguards were implemented to minimise distress or fatigue associated with discussing illness experiences. Participants received detailed information about the study's aims, procedures, and confidentiality measures. Emotional support resources were provided where appropriate. Ethical approval was granted by Northumbria University Ethics Committee (Ref: 13824), the Health Research Authority (IRAS Ref: 257474), and the Newcastle and North Tyneside 1 Research Ethics Committee (Ref: 19‐NE‐0175). The research was authorised by the NHS R&D Department of the hosting Trust (Study No. 9055) and received Caldicott approval. All procedures complied with Good Clinical Practice (GCP) and General Data Protection Regulation (GDPR) standards.

## 3 Results

### 3.1 Sample Characteristics

Most participants were women of working age who were employed, married, and had children, with no notable medical history (Table 1). The majority had been diagnosed with papillary thyroid carcinoma and had undergone either total or partial thyroidectomy, followed by radioactive iodine (RAI) therapy (Table 2).

**TABLE 1:** Sociodemographic characteristics.

Socio‐demographic characteristics	Frequency	Percentage
Gender		
Female	22	88%
Male	3	12%
Other	0	0%
Age		
18–30	4	16%
30–50	11	44%
50–65	9	36%
Over 65	1	4%
Marital status		
Married	15	60%
In relationship	3	12%
Single	3	12%
Engaged	2	8%
Divorced	2	8%
Children	8	32%
No children	6	24%
Adult children	4	16%
Young children	4	16%
Teen children	2	8%
Pregnant	1	4%
Living situation		
Living with partner and children	11	44%
Living with partner	9	36%
Living alone	4	16%
Living with parents	1	4%
Employment		
Full‐time	9	36%
Retired	6	24%
Self employed	4	16%
Part‐time	3	12%
Full‐time study	2	8%
Homemaker	1	4%

*Note:* Sociodemographic characteristics of the study participants. Values are presented as frequencies and percentages. Percentages may not total 100% due to rounding.

**TABLE 2:** Clinical characteristics.

Diagnosis	Frequency	Percentage
PTC (papillary thyroid cancer)	15	60%
FTC (follicular thyroid cancer)	5	20%
Met PTC (metastatic papillary thyroid cancer)	2	8%
Met FTC (metastatic follicular thyroid cancer)	1	4%
PTC and hurtle cell thyroid cancer	1	4%
PTC FTC, and hurtle	1	4%
Treatment		
PT (partial thyroidectomy), then 2nd PT and RAI (radioactive iodine treatment)	9	36%
TT (total thyroidectomy), neck dissection, and RAI	7	28%
TT and RAI	3	12%
TT	3	12%
PT	2	8%
PT, then 2nd PT	1	4%
Past medical history		
None	14	56%
Type 2 diabetes mellitus	2	8%
Depression	3	12%
Other	11	44%

*Note:* Clinical characteristics of the study participants. Values are presented as frequencies and percentages.

Abbreviations: FTC = Follicular Thyroid Cancer; Met = Metastatic; PT = Partial Thyroidectomy; PTC = Papillary Thyroid Cancer; RAI = Radioactive Iodine Treatment; TT = Total Thyroidectomy.

### 3.2 Thematic Analysis

Thematic analysis of the interview transcripts suggested that there were three individual, and two structural, themes when discussing barriers to seeking support. Although the themes describe barriers to support‐seeking, participants did not present these as isolated obstacles. Rather, they emerged through participants' lived experiences of thyroid cancer and reflected how they understood their illness, interpreted available support, and positioned themselves within the healthcare system. The themes therefore capture not only *what* prevented support‐seeking, but *how* participants experienced and made sense of those decisions. They are shown in Table 3:

**TABLE 3:** Barriers to seeking support among thyroid cancer patients.

Broad category	Theme
Domain A: Individual themes	**Theme 1.** ‘I thought I could cope alone’—Self‐sufficiency and emotional resilience
	**Theme 2.** ‘others need it more than me’—Altruistic reluctance and moral comparison
	**Theme 3.** ‘not for people like me’—Age and cancer type as barriers to belonging
Domain B: Structural themes	**Theme 4**. ‘care ends at discharge’—Institutional gaps in post‐treatment support
	**Theme 5**. ‘one‐size Doesn't fit all’—Mismatched and inaccessible support options

These themes are discussed further below.

### 3.3 Theme 1: ‘I Thought I Could Cope Alone’—Self‐Sufficiency and Emotional Resilience

Many participants described experiencing emotional distress as something that should be endured independently. They perceived coping as a personal responsibility, shaping how they interpreted moments of vulnerability. Emotional distress was often seen as transient rather than as an ongoing challenge that required professional or peer help. For some, moments of anxiety or sadness were expected, but they believed that they could cope with these feelings without external intervention. As one participant put it, when asked if they had accessed post‐discharge support:

No, I haven’t. I didn’t feel the need, I had a few days when kind of being a bit overwhelmed a bit, but I sort of got over it and feel fine again.

(TC18)

Another participant acknowledged being aware of support options but dismissed them as unnecessary:

They asked if I wanted to speak with somebody, somebody local, and I think I spoke with her once about either RAI or the process. It wasn’t Macmillan, I just felt a lot of the time that I didn’t need that.

(TC20)

Rather than reflecting an absence of emotional distress, these accounts suggest that participants actively interpreted distress as something that should manage on their own. The decision not to seek support therefore appeared to stem from a personal appraisal that emotional reactions were expected, temporary, and insufficiently serious to justify external assistance. Help‐seeking was framed as unnecessary.

### 3.4 Theme 2: ‘Others Need it More Than Me’—Altruistic Reluctance and Moral Comparison

Participants frequently described an internal tension between recognising their own emotional needs and viewing themselves as insufficiently ill to justify support. Their experience of TC was often framed through comparison with other patients who were perceived to have “more serious” cancers, influencing how they understood their own legitimacy as recipients of care.

And other people need it more, and if I don’t need it I shouldn’t do it.

TC20

This could create a dilemma for those who would have appreciated some support:

I mean it is quite tricky because, in some ways it is like I am healthy and I don’t really want to bother people, maybe they are busy dealing with people with more aggressive forms of thyroid cancer, I just kind of think I don’t want to trouble them.

(NHS02interview3)

These narratives suggest that decisions about support were influenced by moral judgements that were sometimes in tension with personal needs. Participants evaluated their own eligibility for support relative to others, often positioning themselves as less deserving despite acknowledging that additional support might have been beneficial. This comparative reasoning acted as a barrier to engagement.

### 3.5 Theme 3: ‘Not for People Like Me’—Age and Cancer Type as Barriers to Belonging

This theme has been categorised in the individual domain, but is also partly a reflection of a structural factor, that is the support services that were available. Several participants felt alienated from traditional cancer support networks because of their age or the specific nature of TC.

When I was diagnosed, I did a bit of research to see what is out there, I don’t think there is a lot for thyroid cancer patients, but also specifically for young people, it is like children or people that were 40+. And I found it difficult to relate to.

(TC11)

The issue extended beyond the simple availability of services. Participants described a lack of personal identification with existing support opportunities, suggesting that perceived relevance and belonging influenced whether support was considered appropriate. Feeling that services were designed for “someone else” reduced willingness to engage.

### 3.6 Theme 4: ‘Care Ends at Discharge’—Institutional Gaps in Post‐Treatment Support

Participants consistently described a lack of continuity between active treatment and ongoing emotional care. While medical treatment was praised by study participants, emotional and psychosocial support were often perceived as optional extras, provided mainly by external organisations rather than integrated within oncology follow‐up:

When you leave the hospital the care ends, charities step up for the after‐care, for counselling and support.

(TC04)

In some cases, the support at discharge did not even include signposting to other services:

I don’t think I was ever offered any (support). I think I was told the name of the cancer nurse.

(TC20)

Participants interpreted the absence of ongoing contact as signalling that emotional recovery was not part of routine care. Rather than describing isolated service failures, these accounts reflected a broader perception that psychosocial support was peripheral rather than integrated into survivorship.

### 3.7 Theme 5: ‘One‐Size Doesn't Fit All’—Mismatched and Inaccessible Support Options

Even when support was available, some participants found it did not match their personal preferences. For example, group‐based interventions could be perceived as being poorly aligned with participants' personal coping styles, with some finding their own individual support instead:

No, the hospital did suggest it, but what they were offering it was a group thing, so it is not my thing. (…) I shared a body with thyroid cancer, it was taking over me, so I knew that I needed to get healthy and I’m working with a life coach to achieve that, and slowly get me doing things I used to enjoy in the past.

(TC16)

Participants were not necessarily rejecting support itself; instead, they were rejecting forms of support that did not fit their preferences or coping styles. Their accounts suggested that acceptability was as important as availability, with participants often seeking alternative approaches that offered greater personal relevance and autonomy.

## 4 Discussion

### 4.1 Consistency With Existing Literature

Many of these findings were consistent with previous research. Studies have shown that people with TC often downplay distress and make limited use of emotional support services [11, 18] and that beliefs that thyroid cancer is a ‘good’ or ‘curable’ cancer; can make the illness feel less threatening and reduce the perceived need for help [10]. Across cancer groups, patients have minimised their distress to avoid burdening already stretched services [13, 19]. In addition, younger people and those with less visible cancers have often been found to struggle to identify with the broader ‘cancer survivor’ label and/or to feel out of place in mixed‐age environments [20, 21]. Research has demonstrated that patients are more likely to withdraw from services when the mode of delivery does not align with their coping preferences or identity [12, 22] or where they felt that ongoing psychological support is insufficient and difficult to access [23, 24].

### 4.2 Interpretation in Relation to Theoretical Frameworks

While showing that specific findings were consistent with those of previous studies was relatively straightforward, it proved more difficult to find a body of theory that adequately contextualised the themes identified. Discussions of many social phenomena are often sharply divided between those focusing on individual factors and those where structural factors are regarded as key [25]. This was also the case with models to explain a failure to seek help, where none seemed to match the combination of individual and structural factors identified by this research. As a result, it was decided to search for the best fitting individual model, and the best fitting structural model, then to determine whether fusing the two would provide a better framework for understanding the findings.

Several individual models were considered but rejected. For example, the Health Belief Model is primarily concerned with actions that can prevent or manage illness rather than with seeking continuing support following treatment [26]. The Transtheoretical Model posits that there are stages to change [27], but these were not evident among the sample. Instead Leventhal's Common‐Sense Model of Illness Representations (CSM) seemed to provide the best fit with the findings.

CSM proposes that individuals develop cognitive and emotional representations of illness, such as identity, consequences, controllability, timeline and emotional impact, which guide coping responses and influence decisions about seeking support. Within oncology, these representations predict psychological adjustment and engagement with services and are frequently measured using the Brief Illness Perception Questionnaire [28]. Extensions of the CSM to cancer‐specific contexts, including appraisal of recurrence risk, further demonstrate how beliefs and emotions shape ongoing health‐related behaviours [29]. In addition, early‐stage symptom interpretation is influenced by cognitive heuristics that shape how individuals understand bodily changes and determine whether help‐seeking is warranted, offering an additional explanation for delays or underuse of available support [30].

When considering structural level theories, again there were some that were clearly not a good fit with the data. The network episode model emphasises the role of family and friends [31]: however, while social contacts were extremely important in determining whether the experience of survivorship was a positive or a negative one, they did not have a substantial impact on seeking or not seeking help. Stigma theory is sometimes applied to the experience of head and neck cancer [32] but, while scars were a concern for some of the participants, they did not link this concern to the experience of seeking help.

Andersen's Behavioural Model of Health Service Use (ABM) was the chosen structural model. It conceptualises health‐service utilisation as a function of predisposing characteristics (e.g., socio‐demographics and health beliefs), enabling resources (such as service availability, type of services, accessibility and continuity of care), and subjectively perceived or evaluated need [33, 34]. Integrating the belief‐ and emotion‐based elements of the CSM with the largely structural determinants in ABM allows for a more comprehensive understanding of the psychological, contextual and organisational influences on support‐seeking and the barriers to it. This approach aligns with contemporary frameworks of access that highlight the interaction between patient abilities and service characteristics [35, 36]. In other health systems, the presence or absence of insurance is a key factor affecting access to services (see, e.g., [34]). However, even within the UK's system, where healthcare is free at the point of access, this approach offers a coherent conceptual basis for examining the underutilisation of psychosocial support among TC survivors.

It was clear when undertaking this analysis that there was no simple dichotomy between individual and structural themes, or between issues that are incorporated within the CSM and ABM. Although the CSM conceptualises illness beliefs as key determinants of coping behaviour, it also emphasises ongoing self‐regulatory processes in which cognitive and emotional representations influence, and are reshaped by, coping outcomes [37]. In the present study, beliefs that TC was ‘curable’ or ‘not serious enough’ to warrant support were not static traits; rather, they appeared to be reinforced by the organisation of follow‐up care, particularly the limited visibility of psychosocial provision. Similarly, one element of the ABM—subjectively perceived or evaluated need—clearly had links to the beliefs of some participants that others were more in need of services than they were. Predisposing characteristics were also linked to patients' beliefs about whether services were for them. So ‘enabling resources’ was the element of the model that was most clearly focused on structural factors.

Two individual themes (‘I thought I could cope alone’ and ‘Others need it more than me’) and one structural theme (‘Care ends at discharge’) collectively reflected a prioritisation of biomedical over psychosocial need. Participants often interpreted the absence of proactive emotional follow‐up as an implicit signal that distress was peripheral to recovery. In this way, institutional practices influenced how participants interpreted their illness and their place within the healthcare system. This dynamic aligns with broader survivorship literature demonstrating persistent gaps in psychosocial follow‐up after primary treatment [38, 39].

The lack of a clear distinction between either structural or individual domains, or CSM or ABM associated factors, led the authors to examine whether barriers to services could be better understood by combining these different elements. The model that was developed suggested that perceptions of illness seriousness interact with social norms, emotional regulation strategies and perceptions of the services that are available, to shape coping behaviour. Participants often saw TC as manageable or ‘not serious enough’ to justify asking for help. These beliefs, together with a failure of health professionals to provide information about support services, encouraged self‐reliance and the feeling that participants should cope on their own.

These patterns show how illness beliefs and emotional responses work together to shape decisions about support. When they are placed alongside services that are inflexible, limited and poorly signposted, formidable barriers are created. Patients believing that the only services available were in group‐based settings with people older than themselves reinforced the view that they were not suitable to receive support.

The interactions between individual and structural factors are represented in Figure 1:

**FIGURE 1:**
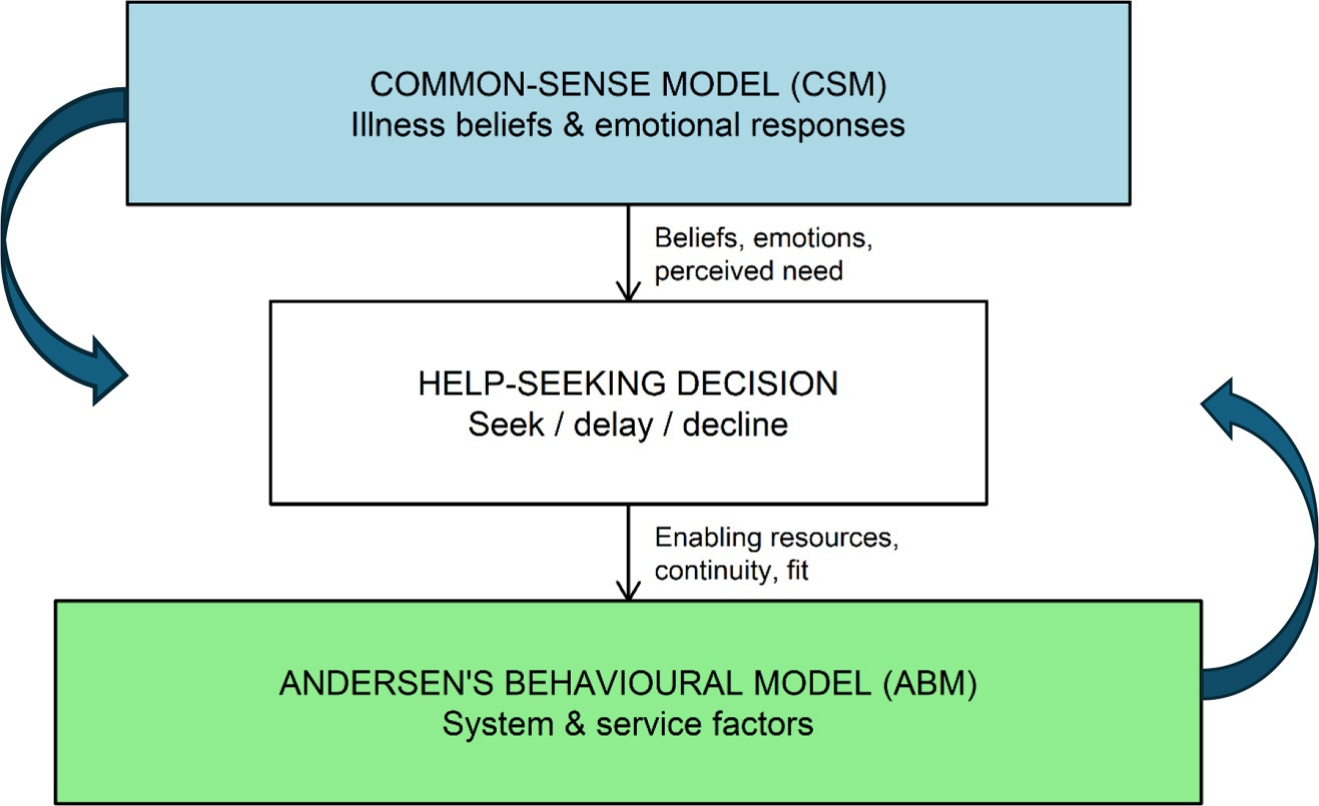
Integrated CSM and ABM framework.

Illness beliefs around self‐sufficiency or low perceived severity were not fixed traits; they were shaped and reinforced by the absence of proactive follow‐up and by service designs that implied emotional needs were peripheral and less important than medical needs. In this way, the study highlights a dynamic feedback loop in which psychological and organisational factors continually shape one another, offering a more integrated and context‐sensitive theoretical account of help‐seeking among TC patients.

These insights show that reluctance to seek help arises not only from how patients understand their illness, but also from moral concerns and identity‐based factors. When individuals feel they do not fully ‘fit’ within the support landscape, or that others need help more than they do, they may step back from services, even when support is available.

### 4.3 Implications for Healthcare Services

At the healthcare level, participants described issues that reflect patterns seen in wider cancer survivorship research, particularly a lack of follow‐up and limited emotional care once active treatment ends. An important contribution of this study is to demonstrate how participants interpreted this lack of psycho‐social follow‐up. Many saw the absence of proactive emotional support as a signal that their distress was not serious or valid enough to require help. Although structural gaps in services are frequently identified [38, 39], the link between service design and personal meaning has received little attention in prior research. This study shows how health‐system practices can unintentionally strengthen the very beliefs that limit help‐seeking. It adds urgency to the need to address the structural gaps in services.

Improving psychosocial support for TC survivors requires approaches that address both personal beliefs and systemic design. Clinicians should recognise that apparent resilience may conceal unmet emotional needs and that self‐reliance often reflects illness representations rather than a genuine absence of distress. Incorporating brief, structured discussions about coping beliefs and emotional well‐being into routine care could help to normalise help‐seeking.

Evidence from distress‐screening research indicates that routine, systematic assessment increases identification of psychosocial concerns and improves referral to appropriate services [40]. Embedding such approaches within TC follow‐up could counter the perception that emotional support is optional or peripheral. Regular survivorship reviews, nurse‐led follow‐up, or structured emotional check‐ins may help normalise help‐seeking, reduce reliance on patient self‐initiation and counter the perception that *‘care ends at discharge’.*

Providing diverse and flexible support options, such as short‐term psychological consultations, digital resources, or tailored one‐to‐one interventions, would ensure accessibility for individuals who prefer more private forms of support. Embedding psychosocial care as a standard component of oncology follow‐up, rather than an optional extra, could reduce inequities and affirm emotional well‐being as integral to recovery.

### 4.4 Implications for Future Research

Future research should build on this integrated framework to explore how psychological and organisational factors jointly predict service engagement across the cancer trajectory. Quantitative and mixed‐methods designs could further test the interactive explanatory power of the combined model.

Longitudinal designs would clarify how beliefs and coping responses evolve from diagnosis through survivorship, while intervention studies could evaluate psychoeducational or clinician‐initiated approaches tailored to TC. Comparative research across other low‐mortality cancers could determine whether similar patterns of self‐reliance and under‐engagement exist, helping to develop cross‐diagnostic psychosocial care frameworks.

### 4.5 Study Limitations

This study has several limitations. As a qualitative enquiry, the findings offer depth rather than statistical generalisability, and participants recruited through clinical and charitable networks may have been more engaged in their care than non‐participants. Retrospective accounts may also be influenced by recall bias, although convergence with prospective interviews strengthens confidence in the themes. The study presents patient perspectives only; future work should incorporate healthcare professionals' views to better understand organisational influences on support provision. Despite these limitations, the analytic richness and diversity of experiences provide valuable insights that are transferable to other, similar survivorship contexts.

## 5 Conclusions

Barriers to psychosocial support among TC survivors arise through the interplay of individual illness beliefs and the structural conditions of routine follow‐up. Feelings of self‐sufficiency, altruism, and the wider social invisibility of the disease often limit help‐seeking, and these tendencies can be quietly sustained by institutional passivity and constrained resources. The CSM and ABM together provide a framework for understanding how these personal and system‐level dynamics interact. A shift towards proactive survivorship care offers a practical way forward. Embedding psychosocial support into routine follow‐up would make emotional well‐being a normal and important part of recovery and help to ensure that survivors' needs were noticed and responded to. Developing such models has the potential not only to improve care in TC, but to inform more equitable and responsive psychosocial pathways across other low‐mortality cancers where emotional needs are often overlooked.

## Author Contributions

Dr Alicja Rogusz led the conception, data collection, analysis, and writing of the manuscript. Dr Jamie Harding provided supervisory guidance, critical feedback, and editorial input. Both authors approved the final version.

## Funding

This research was undertaken as part of a £60,000 doctoral studentship jointly funded by Northumbria University and the Butterfly Thyroid Cancer Trust, with each organisation contributing 50% of the funding. No Grant No. was assigned.

## Conflicts of Interest

Dr Alicja Rogusz received studentship funding jointly from Northumbria University and the Butterfly Thyroid Cancer Trust (BTCT). BTCT also supported participant recruitment and provided input during the design phase of the study. Dr Jamie Harding is employed by Northumbria University. The funders had no role in data collection, data analysis, interpretation of the findings, or preparation of this manuscript. The authors declare no other competing interests.

## Supporting information


Supporting Information S1

## Data Availability

Research data are not shared.
